# The Law Relating to Medical Officers of Health (Outside Metropolis).—III

**Published:** 1903-07-04

**Authors:** J. E. R. Stephens

**Affiliations:** Barrister-at-Law, etc.


### HOSPITAL ADMINISTRATION.

### CONSTRUCTION AND ECONOMICS.

### THE LAW RELATING TO MEDICAL OFFICERS OF HEALTH (OUTSIDE METROPOLIS).-III.

By J. E. R. STEPHENS, Barrister-at-Law, etc.

(*Continued from Page* 214.)

APPOINTMENT OF, BY COUNTY COUNCIL.

SECTION 17 of the Local Government Act, 1888, gives power to the council of any county, if they see fit, to appoint and pay a medical officer of health, or medical officers of health, who shall not hold any other appointment or engage in private practice without express written consent of the council. The County Council, and any district council, may from time to time make and carry into effect arrangements for rendering the services of such officer or officers regularly available in the district of the district council on such terms as to the contribution by the district council to the salary of the medical officer, or otherwise, as may be agreed, and the medical officer shall have within such district all the powers and duties of a medical officer appointed by a district council. So long as such arrangement is in force, the obligation of the district council, under the Public Health Act, 1875, to appoint a medical officer of health shall be deemed to be satisfied without the appointment of a separate medical officer.

APPOINTMENT OF, BY URBAN AND RURAL COUNCIL.

By section 189 of the Public Health Act, 1875, every urban authority must from time to time appoint a fit and proper person to be medical officer of health, and under section 193, every rural authority must from time to time appoint a fit and proper person to be medical officer of health. Section 191 provides that a person shall not be appointed medical officer of health under this Act unless he is a legally-qualified medical practitioner. The same person may, with the sanction of the Local Government Board, be appointed the medical officer of health for two or more districts by the local authorities of such districts, and the Local Government Board shall by order prescribe the mode of such appointment, and the proportions in which the expenses of such appointment, and the salary and charges of such officer shall be borne by such authorities. Any district medical officer of a union may, with the sanction of the Local Government Board and subject to such conditions as the said board may prescribe, be appointed a medical officer of health; and a medical officer of health may exercise any of the powers with which an inspector of nuisances is invested by this Act. In case of illness or incapacity of the medical officer of health, a local authority may appoint and pay a deputy medical officer, subject to the approval of the Local Government Board.

Section 286 of the Public Health Act, 1875, provides that where it appears to the Local Government Board, on any representation made to it that the appointment of a medical officer of health for two or more districts situated wholly or partially in the same county would diminish expense, or otherwise be for the advantage of such districts, the Local Government Board may by order unite such districts for the purpose of appointing a medical officer of health, and may make regulations as to the mode of his appointment and removal by representatives of the authorities of the constituent districts, and as to the meetings from time to time of such representatives, and the proportion in which the expenses of the appointment and of the salary and expenses of such officer are to be borne by such authorities, and as to any other matters (including the necessary expenses of such representatives) which, in the opinion of the said board, require regulation for the purposes of this section; and no other medical officer of health shall be appointed for any constituent district, except as an assistant to the officer appointed for the united districts; provided that no urban district containing a population of 25,000 and upwards or (in the case of a borough) having a separate court of quarter sessions shall be included in any union of districts formed under this section without the consent of the local authority of such district or borough. These may be assigned by the Local Government Board to the district medical officer of any union comprising or coincident with any constituent district such duties in rendering local assistance to the medical officer of health appointed for the united districts as the said board may think fit, and such district medical officer shall receive in respect of any duties so assigned to him, such additional remuneration to be paid by the local authority or authorities of the district or districts within which his duties under this section are performed as those authorities may, with the approval of the Local Government Board, determine.

The County Council may appoint one or more medical officers of health, and an urban or rural authority may utilise his or their services on terms agreed upon between the council and the local authority, in which case the local authority need not appoint a separate medical officer. The same person may, with the sanction of the Local Government Board, be medical officer of health for two or more districts. Medical and other officers may be specially appointed when the Local Government Board have issued regulations on the outbreak of a formidable epidemic. Section 134 of the Public Health Act, 1875, enacts that whenever any part of England appears to be threatened with or is affected by any formidable epidemic, endemic, or infectious disease, the Local Government Board may make, and from time to time alter and revoke, regulations for all or any of the following purposes, namely:—(1) For the speedy interment of the dead, and (2) for house-to-house visitation, and (3) for the provision of medical aid and accommodation, for the promotion of cleansing, ventilation, and disinfection, and for the guarding against the spread of disease; and may by order declare all or any of the regulations so made to be in force within the whole or any part or parts of the district of any local authority, and to apply to any vessels, whether on inland waters or on arms or parts of the sea within the jurisdiction of the Lord High Admiral of the United Kingdom, or the Commissioners for executing the office of the Lord High Admiral for the time being, for the period in such order mentioned, and may by any subsequent order abridge or extend such period. The Public Health Act, 1896, without prejudice to the generality of the powers conferred by the above section, specifies certain matters which may be dealt with by the regulations. Section 136 of the Act of 1875 enacts that the local authority of any district within which or part of which regulations so issued by the Local Government Board are declared to be in force shall superintend and ee to the execution thereof, and shall appoint and pay such medical or other officers or persons, and do and provide such acts, matters, and things as may be necessary for mitigating any such disease, or for superintending or aiding in the execution of such regulations, or for executing the same, as the case may require. Moreover, the local authority may from time to time direct any prosecution or legal proceedings for or in respect or wilful violation or neglect of any such regulation.

### Certificate of Medical Officer of Health.

*As to Purification of House.*—It is enacted by section 46 of the Public Health Act, 1875, that where on the certificate of the medical officer of health or of any two medical practitioners, it appears to any local authority that any house or part thereof is in such a filthy or unwholesome condition that the health of any person is affected or endangered thereby, or that the whitewashing, cleansing or purifying of any house or part thereof would tend to prevent or check infectious disease, the local authority shall give notice in writing to the owner or occupier of such house or part thereof to whitewash, cleanse, or purify the same as the case may require. If the person to whom notice is so given fails to comply therewith within the time therein specified, he shall be liable to a penalty not exceeding 10s. for every day during which he continues to make default; and the local authority may, if they think fit, cause such house or part thereof to be whitewashed, cleansed or purified, and may recover in a summary manner the expenses incurred by them in so doing from the person in default.

Section 120 of the same Act provides that where any local authority are of opinion, on the certificate of their medical officer of health or of any other legally-qualified medical practitioner, that the cleansing and disinfecting of any house or part thereof, and of any articles therein likely to retain infection would tend to prevent or check infectious disease, it shall be the duty of such authority to give notice in writing to the owner or occupier of such house or part thereof requiring him to cleanse and disinfect such house or part thereof and articles within a time specified in such notice. If the person to whom notice is so given fails to comply therewith, he shall be liable to a penalty of not less than 1s. and not exceeding 10s. for every day during which he continues to make default; and the local authority shall cause such house or part thereof and articles to be cleansed and disinfected, and may recover the expenses incurred from the owner or occupier in default in a summary manner. Where the owner or occupier of any such house or part thereof is from poverty or otherwise unable, in the opinion of the local authority, effectually to carry out the requirements of this section, such authority may, without enforcing such requirements on such owner or occupier with his consent cleanse and disinfect such house or part thereof and articles, and defray the expenses thereof.

*As to Offensive Trade.*—Where any candle-house, malting-house, melting-place, or soap-house, or any slaughter-house, or any building or place for boiling offal or blood, or for boiling, burning or crushing bones, or any manufactory, building, or place used for any trade, business, process, or manufacture, causing effluvia, is certified to any urban authority by their medical officer of health, or by any two legally qualified medical practitioners, or by any ten inhabitants of the district, such urban authority shall direct complaint to be made before a justice, who may summon the person by or on whose behalf the trade so complained of is carried on, to appear before a court of summary jurisdiction. The court shall inquire into the complaint, and if it appears to the court that the business carried on by the person complained of is a nuisance, or causes any effluvia which is a nuisance or injurious to the health of any of the inhabitants of the district, and unless it be shown that such person has used the best practicable means for abating such nuisance, or preventing or counteracting such effluvia, the person so offending (being the owner or occupier of the premises, or being a foreman or other person employed by such owner or occupier) shall be liable to a penalty not exceeding £5 nor less than 40s., and on a second and any subsequent conviction to a penalty double the amount of the penalty imposed for the last preceding conviction, but the highest amount of such penalty shall not in any case exceed the sum of £200. But the court may suspend its final determination on condition that the person complained of undertakes to adopt, within a reasonable time, such means as the court may deem to be practicable and order to be carried into effect for abating such nuisance, or mitigating or preventing the injurious effects of such effluvia, or if such person gives notice of appeal to the Court of Quarter Sessions in manner provided by this Act. Any urban authority may, if they think fit, on such certificate as is in this section mentioned, cause to be taken any proceedings in any superior court of law or equity against any person in respect of the matters alleged in such certificate (section 114).

*As to Retention of Dead Bodies.*—Under section 8 of the Infectious Disease (Prevention) Act 1890 no person without the sanction in writing of the medical officer of health or of a registered medical practitioner, shall retain unburied elsewhere than in a public mortuary or in a room not used at the time as a dwelling-place, sleeping-place, or workroom, for more than 48 hours, the body of any person who has died of any infectious disease. And it is also provided by section 142 of the Public Health Act 1875 that where the body of one who has died of any infectious disease is retained in a room in which persons live or sleep, or any dead body which is in such a state as to endanger the health of the inmates of the same house or room is retained in such house or room, any justice may on a certificate signed by a legally qualified medical practitioner, order the body to be removed, at the cost of the local authority, to any mortuary provided by such authority, and direct the same to be buried within a time to be limited in such order; and unless the friends or relations of the deceased undertake to bury the body within the time so limited, and do bury the same, it shall be the duty of the relieving officer to bury such body at the expense of the poor rate, but any expense so incurred may be recovered by the relieving officer in a summary manner from any person legally liable to pay the expense of such burial. Any person obstructing the execution of an order made by a justice under this section shall be liable to a penalty not exceeding £5.

Section 9 of the Infectious Disease (Prevention) Act 1890 provides that if any person shall die from any infectious disease in any hospital or place of temporary accommodation for the sick, and the medical officer of health, or any other registered medical practitioner, certifies that in his opinion it is desirable, in order to prevent the risk of communicating any infectious disease or of spreading infection, that the body shall not be removed from such hospital or place except for the last-mentioned purpose; and when the body is taken out of such hospital for that purpose it shall be forthwith carried or taken direct to some cemetery or place of burial, and shall be forthwith there buried, and any person wilfully offending against this section shall be liable to a penalty not exceeding £10. Nothing in this Act shall prevent the removal of any dead body from any hospital or temporary place of accommodation for the sick to any mortuary, and such mortuary shall, for the purposes of this section, be deemed part of such hospital or place as aforesaid. By section 10 of the same Act it is provided that where the body of any person who has died from any infectious disease remains unburied elsewhere than in a mortuary, or in a room not used at the time as a dwelling-place, sleeping-place, or workroom, for more than 48 hours after death without the sanction of the medical officer of health or of a registered medical practitioner, or where the dead body of any person is retained in any house or building so as to endanger the health of the inmates of such house or building, or of any adjoining or neighbouring house or building, any justice may, on the application of the medical officer of health, order the body to be removed at the cost of the local authority to any available mortuary, and direct the same to be buried within a time to be limited in the order; and any justice may, in the case of the body of any person who has died of any infectious disease, or in any case in which he shall consider immediate burial necessary, direct the body to be so buried. Unless the friends or relatives of the deceased undertake to bury and do bury the body within the time limited by such order, it shall be the duty of the relieving officer of the relief district from which the body has been removed to the mortuary, or in which the body shall be, if it has not been so removed, to bury such body, and any expense so incurred may be charged by the relieving officer in his accounts, and may be recovered by the board of guardians in a summary manner from any person legally liable to pay the expenses of such burial.

